# An inactivating human TRPC6 channel mutation without focal segmental glomerulosclerosis

**DOI:** 10.1007/s00018-023-04901-w

**Published:** 2023-08-24

**Authors:** Lilas Batool, Krithika Hariharan, Yao Xu, Mario Kaßmann, Dmitry Tsvetkov, Björn-Oliver Gohlke, Sylvia Kaden, Manfred Gossen, Bernd Nürnberg, Andreas Kurtz, Maik Gollasch

**Affiliations:** 1 BIH Center for Regenerative Therapies (BCRT), Charité-Universitätsmedizin Berlin, Augustenburger Platz 1, 13353 Berlin, Germany; 2 Fraunhofer-Institute for Biomedical Engineering (IBMT), Fraunhofer Project Center for Stem Cell Process Engineering, Würzburg, Germany; 3 Klinik und Poliklinik für Innere Medizin D-Geriatrie, Universitätsmedizin Greifswald, Ferdinand-Sauerbruch-Straße, Greifswald, Germany; 4 Department of Information Technology, Science-IT, Charité-Universitätsmedizin Berlin, Berlin, Germany; 5 Electron Microscopy Core Facility, German Cancer Research Center, Heidelberg, Germany; 6 Institut für Aktive Polymere, Hereon TeltowAbteilung Stammzellmodifikation und Biomaterialien, Teltow, Germany; 7 Department of Pharmacology, Experimental Therapy and Toxicology, Institute of Experimental and Clinical Pharmacology and Pharmacogenomics, University of Tübingen, Tübingen, Germany; 8 Biomedical Data and Bioethics, Fraunhofer-Institute for Biomedical Engineering (IBMT), Berlin, Germany; 9 Klinik für Nephrologie und Internistische Intensivmedizin, Charité-Universitätsmedizin Berlin, Berlin, Germany

**Keywords:** FSGS, TRPC6, Podocyte, Calcium, Truncated mutation

## Abstract

Transient receptor potential cation channel-6 (*TRPC6*) gene mutations cause familial focal segmental glomerulosclerosis (FSGS), which is inherited as an autosomal dominant disease. In patients with *TRPC6*-related FSGS, all mutations map to the N- or C-terminal TRPC6 protein domains. Thus far, the majority of TRPC6 mutations are missense resulting in increased or decreased calcium influx; however, the fundamental molecular mechanisms causing cell injury and kidney pathology are unclear. We report a novel heterozygous TRPC6 mutation (V691Kfs*) in a large kindred with no signs of FSGS despite a largely truncated TRPC6 protein. We studied the molecular effects of V691Kfs* TRPC6 mutant using the tridimensional cryo-EM structure of the tetrameric TRPC6 protein. The results indicated that V691 is localized at the pore-forming transmembrane region affecting the ion conduction pathway, and predicted that V691Kfs* causes closure of the ion-conducting pathway leading to channel inactivation. We assessed the impact of V691Kfs* and two previously reported TRPC6 disease mutants (P112Q and G757D) on calcium influx in cells. Our data show that the V691Kfs* fully inactivated the TRCP6 channel-specific calcium influx consistent with a complete loss-of-function phenotype. Furthermore, the V691Kfs* truncation exerted a dominant negative effect on the full-length TRPC6 proteins. In conclusion, the V691Kfs* non-functional truncated TRPC6 is not sufficient to cause FSGS. Our data corroborate recently characterized TRPC6 loss-of-function and gain-of-function mutants suggesting that one defective *TRPC6* gene copy is not sufficient to cause FSGS. We underscore the importance of increased rather than reduced calcium influx through TRPC6 for podocyte cell death.

**Supplementary Information:**

The online version contains supplementary material available at 10.1007/s00018-023-04901-w.

## Introduction

Focal segmental glomerulosclerosis (FSGS) causes nephrotic syndrome and can promptly progress to end-stage renal disease [1]. FSGS is characterized by sclerosis in some (focal) but not in all glomeruli, while solidification of glomerular tuft and lesions demonstrates segmental distribution leading to proteinuria [2]. Adult-onset C1q nephropathy is an FSGS form characterized by mesangial C1q deposits that appear to be a non-specific marker of increased mesangial transport in the setting of glomerular proteinuria [3, 4]. Podocyte injury is the earliest morphological hallmark of FSGS supporting the notion that FSGS is a podocyte disorder. Genetic linkage studies have identified at least 27 genes accountable for dominant or recessive familial FSGS forms [5]. The genes encode proteins expressed in podocytes and are involved in a wide range of podocyte functions. Heterozygous mutations in transient receptor potential (TRPC) cation channel, subfamily C, member 6 (*TRPC6*) cause late and early onset of FSGS. TRP channels are homo or/and heterotetramers containing six transmembrane segments (S1–S6) with cytoplasmic N- and C-terminal tails. The S1–S4 segments and cytoplasmic N- and C-termini are significant for gating and interaction with ligands proteins; the S5 and S6 segments and connecting pore loop form the cation-conducting pore [6, 7].

Podocyte TRPC6 mediates calcium entry on hormonal stimulation via phospholipase C (PLC) upon G protein-coupled receptor stimulation resulting from the breakdown of phosphatidylinositol-4,-5-bisphosphate (PIP_2_), with the generation of diacylglycerol (DAG) directly activating the TRPC6 channel [8]. TRPC6 colocalizes and physically interacts with slit diaphragm podocin and nephrin, and podocyte cytoskeleton, forming signaling complexes that maintain the filtration barrier integrated [9]. Most of the identified *TRPC6* mutations were missense mutations [9–21], which based on functional studies are categorized into gain-of-function (GOF; R68W, G109S, N110H, P112Q, M132T, Q134P, N143S, R175Q, H218L, S270T, A404V, Q889K, R895C, and E897K) or loss-of-function (LOF; N125S, L395A, G757D, L780P, and R985L) mutants. Notably, both GOF and LOF mutations in the TRPC6 channel are believed to evoke the same FSGS disease phenotype [19, 22]. However, how these diverse mutants all cause a similar phenotype is poorly understood.

The mutants are mapped to the terminal domains of the TRPC6 protein. Ten N-terminal missense mutations (G109S, N110H, P112Q, M132T, N125S, N143S, R175Q, H218L, S270T, and A404V) locate in or near ankyrin repeats and at adjacent lipid/trafficking domain. The ankyrin domains are responsible for the self-association of TRPC homomers [23], whereas the lipid-binding domain binds DAG and participates in the translocation of the channel to the plasma membrane [24]. Most of the GOF mutations of the TRPC6 that cause FSGS are located in the intracellular cytosolic domain architecture of TRPC6 [25]. The L780P mutation is located near the EWKFAR motif, a highly conserved proline-rich motif, whereas four additional mutations (K874X, Q889K, R895C, and E897K) mapped to a predicted coiled-coil domain at the C-terminus are predicted to be GOF mutants [26]. One of the LOF mutations (G757D) locates in the TRP domain next to the L780P mutation and hampers a compact structure of the tetrameric channel, affecting overall activity, which is in line with TRPC6 G757D being a dominant negative mutant. Data suggest that LOF is a direct cause of FSGS in humans [19]. This obvious diversity in the molecular phenotypes of these mutants has become a perplexing enigma for understanding the mechanisms of TRPC6 in kidney disease.

We report a new TRPC6 mutation Val691Lysfs* (V691Kfs*) in a large kindred that induces a truncated TRPC6 protein lacking the C-terminal coiled-coil domain, a highly conserved region essential for TRPC6 channel regulation. Functional studies revealed that the mutant TRPC6 channel is non-functional and inactivated, resulting in a complete lack of calcium influx, which is consistent with the LOF phenotype. However, affected members had no FSGS. Our findings demonstrate that TRPC6 channel inactivation due to LOF mutants or inactivated mutants does not cause FSGS in humans. Furthermore, our findings not only augment the mutational spectrum of TRPC6-related FSGS but also question the current understanding of the molecular mechanisms leading to FSGS.

## Materials and methods

### Site-directed mutagenesis

Plasmid DNA coding for five different TRPC6 mutants with C-terminally fused YFP (P112Q, M132T, G757D, L395A, and LFW679AAA) were described previously [19]. To achieve an inducible expression of the different TRCP6 mutants, site-directed mutagenesis was performed by using an XLone plasmid with Tet-On 3G system [27] that includes PiggyBac transposase with inverted terminal repeats, two promoters, and poly(A) sequences. In this plasmid the first promoter, TRE3G promoter controls the expression of elements inserted into the multiple cloning sites (MCS), i.e., TRPC6 wild type (WT), P112Q (GOF), V691Kfs* (truncated), and G757D (LOF) fused with a yellow fluorescent protein (YFP), located downstream of the promoter. The second promoter, the EF-1α promoter, controls the expression of a resistance gene for the drug blasticidin (Bsd) and the Tet-On 3G transactivator protein (Fig. 3a). This system utilizes a doxycycline-binding transactivator protein and a promoter to regulate gene expression. The expression level of the gene of interest under the pTRE3G promoter is regulated by changes in doxycycline (Dox) concentration. To insert the TRPC6-YFP gene sequence from the original plasmid (Addgene# 21084 and mutants, Table S1) in the recipient XLone vector (Addgene #96930), both the vector and insert were digested with *KpnI* and *BsrGI* (New England BioLabs, Frankfurt, Germany) restriction enzymes and ligated using Quick Ligation™ Kit (New England BioLabs, Frankfurt, Germany). After quick ligation using 50 ng of vector and a threefold molar excess of insert, the ligation product was transformed into *E. coli* Top 10 F (Fisher Scientific, Darmstadt, Germany) competent cells following standard protocols. To verify the correct mutation sequences for each plasmid, Sanger sequencing was performed using the primers listed in Table S1. Snap gene® (version 3.3.4, GSL Biotech LLC) cloning software was used to analyze the DNA sequences. After plasmid integrity was confirmed, large-scale DNA preparation was performed using NucleoBond Xtra Maxi Kits (Macherey-Nagel, Duren, Germany).

### Cell culture and transfection

HeLa cells were grown in Dulbecco’s Modified Eagle Medium GlutaMAX™ (DMEM; Fisher Scientific, Darmstadt, Germany) supplemented with 10% fetal calf serum (FCS; Sigma-Aldrich, Darmstadt, Germany) and 100 U/mL Penicillin–Streptomycin (Biochrom, Berlin, Germany) in the presence of 5% CO_2_ at 37 °C. For the experiment, cells were seeded in 24-well plate (Stem cell technologies, Cologne, Germany). When cells were 70–80% confluent, they were transfected with XLone TRPC6-YFP plasmids using Lipofectamine 3000 (Invitrogen, Darmstadt, Germany) according to the manufacturer’s manual. A total of 500 ng plasmid DNA containing PiggyBac (PB) transposase, coding for wild-type or TRPC6 mutants C-terminally fused with YFP fluorescent protein was added to 50 μL OptiMEM (Fisher Scientific, Darmstadt, Germany) with 1.5 μL of P3000 reagent. The second solution of 1.5 μL Lipofectamine 3000 reagent in 50 μL OPTIMEM was made. Both solutions were combined and incubated for 5 min at room temperature (RT), and then 100 µL of the mixture was added per well. After overnight incubation at 37 °C, fresh DMEM media supplemented with 0.5 µg/mL of doxycycline were applied to cells. After one day, induced YFP expression was analyzed using Operetta high-content imaging system (Perkin Elmer). After 24 h of transfection, cells were selected with blasticidin for five days. Antibiotic-resistant cells were then imaged regularly every 24 h.

For the co-transfection experiments, cells were transfected with 2 µg mixture of plasmid DNAs, coding for wild-type TRPC6 C-terminally fused to YFP and mCherry and TRPC6 mutants C-terminally fused to mCherry and YFP. The individual mixtures enabling 1:1 expression of YFP—as well as mCherry-fused protein were determined using the double-positive (mCherry and YFP) sorting strategy by Cell Sorter—Aria II “Calliope” technique. Based on the fluorescence intensities obtained from cells expressing an intramolecularly fused YFP-mCherry tandem protein by microscopy images allowed us to identify a mixture of plasmid DNA reproducibly enabling the expression of equimolar amounts of both channel proteins as a basis for the validity of the co-expression experiments.

### Flow cytometry analysis and fluorescence-activated cell sorting

To analyze YFP expression, cells were treated with doxycycline (Dox) 24 h before FACS analysis. Cells were dissociated into single cells with 0.5 mM Trypsin/ETDA in DPBS at 37 °C for 5 min. Cells were centrifuged at 300*g* for 5 min and the pellet was re-suspended into DPBS with 2% FCS. For flow cytometry analysis, data were collected using MACSQuant VYB Flow Cytometer (Miltenyi Biotec GmbH, Version 2) and analyzed using FlowJo software (FLOWJO LCC). For fluorescence-activated cell sorting, the flow cytometer Aria II “Calliope” (BD Biosciences) was used by collecting only the cell population with the highest YFP expression. In both analyses, FACS gating was based on un-transfected and untreated cells as a control. The results shown are from three independent experiments performed with three technical replicates for each mutant.

### Single-cell fluorescence measurements

Ca^2+^ increase was measured intracellularly in HeLa cells as previously described [28, 29]. Ca^2+^ imaging was performed on transgenic HeLa cells (1 × 10^4^ per well) that were seeded on 96-well plates (Perkin Elmer LLC Cell Carrier-96 ultra microplates). Cells were administered doxycycline (Dox, 0.5 mg/mL) 24 h before the start of the experiment to express YFP signals indicating the induction of TRPC6 mutants. On the same day, cells were incubated with the Ca^2+^ indicator fluo-4 AM (10 µmol/L; Fisher Scientific, Darmstadt, Germany) and pluronic acid (0.005%, w/v; Fisher Scientific, Darmstadt, Germany) for 60 min at room temperature (RT) in Ca^2+^ free Hank’s solution (Sigma-Aldrich, Darmstadt, Germany) [28, 29]. Afterward, cells were washed with HEPES-buffered physiological saline solution (HEPES-PSS) for 10 min at RT. Transgenic HeLa cells were imaged in HEPES-PSS solution that had the composition of (mM): 134 NaCl, 6 KCl, 1 MgCl_2_, 2 CaCl_2_, 10 glucose, and 10 HEPES (pH 7.4, NaOH). Nipkow disk-based UltraView LCI confocal scanner (Perkin Elmer, Waltham, MA, USA) linked to a fast digital camera (Hamamatsu Photonics Model C4742-95-12ERG, 1344 × 1024 active pixel resolution, 6.45 μm square pixels, Hamamatsu Photonics Co., Hamamatsu, Japan) was used for recording the images. The confocal system was mounted on an inverted Eclipse Ti microscope with a 40× oil-immersion objective (NA 1.3, Nikon Inc., Melville, NY, USA). Images were obtained by illumination with an argon laser at 488 nm, and recording all emitted light above 515 nm. [Ca^2+^] analyses were performed offline using the UltraView Imaging Suite software (Perkin Elmer, Waltham, MA, USA).

We measured intracellular Ca^2+^ increases in control, TRPC6 wild-type, and TRPC6 mutants in response to carbachol (100 µM; Sigma-Aldrich, Darmstadt, Germany), which stimulates endogenously expressed muscarinic acetylcholine receptors in HeLa cells. Receptor activation of the cells by carbachol is known to cause activation of phospholipase C and subsequent breakdown of phosphatidylinositol-4,5-bisphosphate generating inositol trisphosphate and diacylglycerol, activating TRPC6 channel [8]. The TRPC6 activity was also measured against 1,2-dioctanoyl-*sn*-glycerol (DOG) (100 µM; Avanti Polar Lipids, Alabaster, AL), a direct activator of TRPC6. Additionally, the effect of TRPC6 channel blockers SAR7334 (100 nM; TOCRIS, Wiesbaden-Nordenstadt, Germany) and SH045 (100 nM) as previously described [30, 31] was analyzed in transfected cells by pre-incubating cells with either of the channel blockers for 10 min before treating them with carbachol and DOG. The entire area of each image was analyzed to detect intracellular Ca^2+^ changes. Ca^2+^ changes were defined as local fractional fluorescence increase (*F*/*F*_0_) above the noise level of 1.5. The maximal amplitude of carbachol/DOG-induced peak fluorescence was normalized by the initial fluorescence value (*F/F*_0_) and considered as an index of the total peak Ca^2+^ influx.

### Patch-clamp measurements

Standard whole-cell recordings were performed at room temperature using an Axopatch 200B (Axon Instruments/Molecular Devices, Sunnyvale, CA) or an EPC7 (List, Darmstadt, Germany) amplifier under the control of Clampex software (Molecular Devices, Sunnyvale, CA) and digitized at 5 kHz (ramp protocol) or 2 kHz (continuous recording at −60 mV) using a Digidata 1440A digitizer (Axon CNS, Molecular Devices) [19]. Pipettes were made from borosilicate glass capillary tubes and had resistances of 2–9 MΩ. Time courses of inward currents at a holding potential of −60 mV were recorded from transfected HEK293 cells during the extracellular application of carbachol and gadolinium chloride (each at 100 µM). Voltage ramps were obtained from −100 to + 100 mV (1400 ms duration). Pipettes for whole-cell recordings were filled with a solution of 130 mM CsCH_3_O_3_S, 10 mM CsCl, 2 mM MgCl_2_, 3 mM Na_2_ATP, and 10 mM HEPES (pH 7.2 with CsOH). The standard bath solution was composed of 135 mM NaCl, 5 mM KCl, 2 mM CaCl_2_, 1 mM MgCl_2_, 10 mM glucose, and 10 mM HEPES (pH 7.4 with NaOH).

### Western blotting

Cells were washed with ice-cold PBS and then lysed with RIPA buffer (Sigma) containing 1× protease inhibitor (Roche). Cell lysate was harvested, sonicated (2 min), and centrifuged at 16,000*g* for 20 min. The protein content in the resulting supernatant was quantified by Bradford Assay (Pierce TM BCA Protein Assay Kit, ThermoFisher Scientific) according to the manufacturer’s protocol. Each sample was prepared by mixing 10 µg of protein, 2× Laemmli Buffer (Bio-Rad), and 100 mM DTT (ThermoFisher) to make a total of 30 µl. Samples were heated at 70 °C for 10 min and loaded into a pre-casted 10-well Bolt 12% Bis–Tris mini protein gel (Invitrogen). The PageRuler 10–180 kDa (ThermoFisher Scientific) was used as the protein size marker. Electrophoresis in 1X NUPAGE MOPS SDS running buffer (Invitrogen) was performed, followed by transferring the samples to 0.45 μm nitrocellulose membranes (GE Healthcare) by wet blotting in NuPAGE transfer buffer (Invitrogen). The membrane was blocked by Intercept T20 Antibody Diluent (LI-COR Bioscience) for 1 h at room temperature on the shaker and incubated with rabbit anti-TRPC6 (Merck Millipore 1:300) antibody and Rabbit ß-actin (New England Biolabs GmbH, clone 13E5, 1:1000) in Intercept T20 antibody diluent (LI-COR Biosciences) at 4 °C overnight. Membranes were washed twice with Tris-buffer saline 0.1% Tween 20 (TBS-T) for 10 min and once with Tris-buffer saline (TBS). The samples were incubated with IRDye 800 CW donkey anti-mouse IgG (LI-COR Biosciences, 1:10,000) and IRDye 680 RD donkey anti-rabbit IgG (LI-COR Biosciences, 1:10,000) for 1 h at room temperature under shaking. After washing twice in TBS-T for 5 min, membranes were kept in Tris-buffered saline (TBS) and visualized with the ChemiDoc MP Imaging system (Bio-Rad).

### Statistical analyses

Measured values are presented as means ± SEMs. Microsoft Office Excel and GraphPad Prism (versions 5 and 8, 0, 1) were used for statistical analyses and the creation of graphs. The signal intensities were calculated as differences between baseline and peak and normalized with wild-type (WT) TRPC6. The source data are available on request.

### Patient data

The index patient is a 45-year-old woman. The patient was entered into the study after having given written informed consent.

## Results

### Clinical history and pedigree analysis

**Fig. 1:**
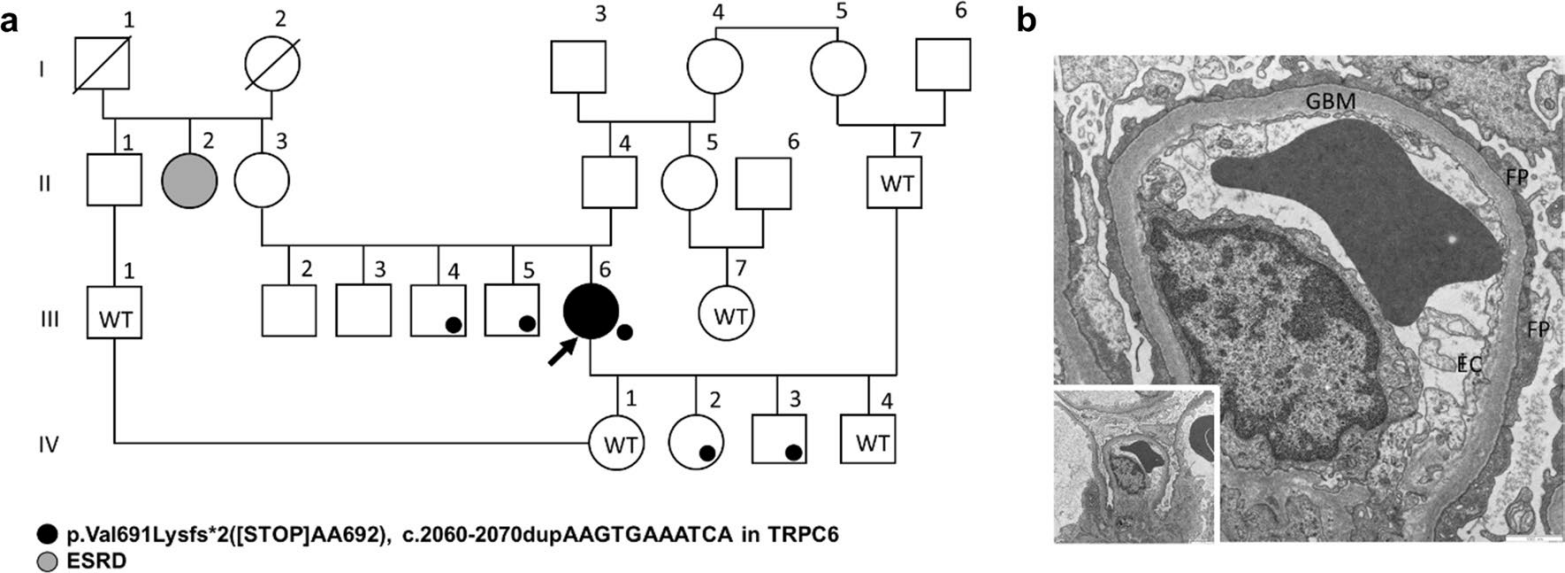
**a** Pedigree of Large kindred with the novel p.Val691Lysfs*2 (V691Kfs*) ([STOP]AA692) mutation. Small closed circle, V691Kfs*; *WT* wild-type genotype. *ESRD* end-stage renal disease. **b** Electron micrograph of the glomerulus of index patient III-6 showing loss of foot processes (FP) of the podocytes; glomerular basement membranes (GBM) are of regular width and without structural alterations. Endothelial cells (EC) are typical. Magnification: 1000 nm

We identified a woman with a novel dominant heterozygous TRPC6 mutation (V691Kfs*). The index patient is a 45-year-old woman with nephrotic syndrome who developed end-stage renal disease (ESRD) within less than 3 years. She first presented to the nephrology clinic in 2018 with bilateral lower limb edema and an altered renal profile. Her serum creatinine was 0.9 mg/dL with low serum albumin of 20 g/L and proteinuria in the nephrotic range, with protein creatinine index (UPCI) of 34 g/g in the urine. Renal biopsy showed no glomerular lesions by light microscopy. Electron microscopy of renal samples revealed loss of foot processes of podocytes, while glomerular basement membranes showed no structural alterations (Fig. 1b). No immune deposits in the form of electron-dense deposits was found in the mesangium or in basement membranes. The clinical and pathologic findings are consistent with minimal change disease (MCD). Despite treatment with prednisolone, serum creatinine and proteinuria increased to 3.3 mg/dL and 26 g/g, respectively, within 3 months. Partial remission was achieved with cyclophosphamide within 6 months (creatinine 1.3 mg/dL, proteinuria 9.6 g/g, albuminuria 8.3 g/g). The patient again failed to appear for follow-up and sought alternative medications for her renal insufficiency. Compliance was reduced due to depressive episodes. In 2020, she presented again to the emergency department with an obvious nephrotic syndrome, severe edema, and ascites. Her serum creatinine was 5.0 g/dL (eGFR CKD-EPI < 15 mL/min per 1.73 m^2^), serum albumin 10 g/dL, UPCI 18 g/g, and urinary albumin creatinine index 8.5 g/g. A dialysis fistula was constructed. There are four people in the pedigree (Fig. 1a), who share the same TRPC6 mutation (V691Lfs*) besides the proband. Two siblings, aged 23 (III-4) and 39 (III-5) years younger than proband at the presentation time, were without kidney disease and had normal renal function. In Sibling III-4, serum creatinine was 0.8 mg/dL, serum albumin 45 g/L, and urinary protein creatinine index (UPCI) 55 mg/g. In sibling III-5, serum creatinine was 0.78 mg/dL, serum albumin 42.3 g/L, UPCI 58 mg/g, and urinary albumin creatinine index 4 mg/g. All these values are in the normal range. Two other brothers refused genetic testing since they had no kidney disease. Similarly, two out of four children (IV-2 and IV-3) of the proband harbored the V691Kfs* mutation and none had the renal disease. In child IV-2, serum creatinine was 0.7 mg/dL, serum albumin 48.4 g/L, UPCI 58 mg/g, and urinary albumin creatinine index 4.8 mg/g. In child IV-3, serum creatinine was 0.4 mg/dL, UPCI 55 mg/g, and urinary albumin creatinine index 3 mg/g. All these values are in the normal range. The patient’s aunt (II-2) was reportedly receiving dialysis (Fig. 1a); however, her underlying renal disease is unknown, and a request for genetic testing was denied.

### Structural evaluation of the truncated tetrameric TRPC6 mutant

**Fig. 2:**
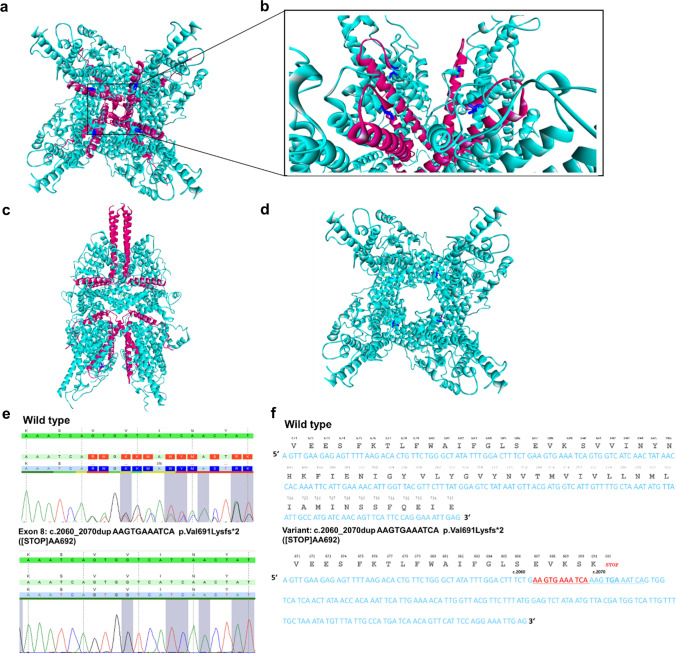
Tridimensional cryo-EM structure of tetrameric TRPC6 (PDB: 6UZ8) indicating novel truncated p.Val691Lysfs*2 (V691Kfs*) ([STOP]AA692) protein in exon 8. **a** View from the extracellular side, representing four monomeric TRPC6 subunits forming the ion channel complex. **b** Close-up view of the helices shows an exchange of Valine to Lysine at amino acid position p.691 and in a stop codon at p.692 making a truncated protein. **c** Full homology view parallel to the membrane. The location of the V691 protein is indicated in dark blue color and red color illustrates the truncated part of the closed channel. **d** Truncation results in complete loss of the C-terminal helices. **e** Verification of c.2060_2070dup AAGTGAAATCA variant compared with wild-type TRPC6 sequence.** f** Comparison of variant sequence with nucleotide insertion and addition of stop codon at p.692, with wild-type TRPC6 sequence

The structural consequences of the new mutation were studied using tridimensional cryo-electron microscopy (cryo-EM) [32]. This structure was solved at a resolution of 4.36 Å. The structure (6UZ8) from the protein data bank (PDB, EMD-20953) [32] was used to examine and highlight the respective positions and effects of the novel truncated mutant. The tridimensional morphology is displayed from the extracellular side (Fig. 2a) and a side view with a typical bell shape structure (Fig. 2c). The position of novel FSGS-related truncated mutation was analyzed in this structure. Genetic testing of TRPC6 showed a heterozygote frame shift DNA mutation with the insertion of AAGTGAAATCA amino acid sequence at the codon position ca. 2060–2070 results in the exchange of valine to lysine at amino acid position p.691 and a stop codon at p.692 making a truncated protein (Fig. 2d). V691 is located on the top of the pore at the transmembrane domain (TMD) S6 that gates the ion conductance of the channel lining the pore lumen in the TRPC6 structure (Fig. 2a, b and Supplementary Fig. 1a). Truncation of the protein due to stop codon results in complete loss of the C-terminus helices at the top of the complex (Fig. 2d and supplementary Fig. 1b, c), which together with the valine to lysine exchange at position 691 is expected to suppress ion permeability through the TRPC6 channel pore. The reported mutation is novel, as it has not been reported in any dbSNP or exome sequencing databases of normal control, and has not been reported previously in *TRPC6*.

### TRPC6-inducible overexpression elucidates mutant functionality

**Fig. 3:**
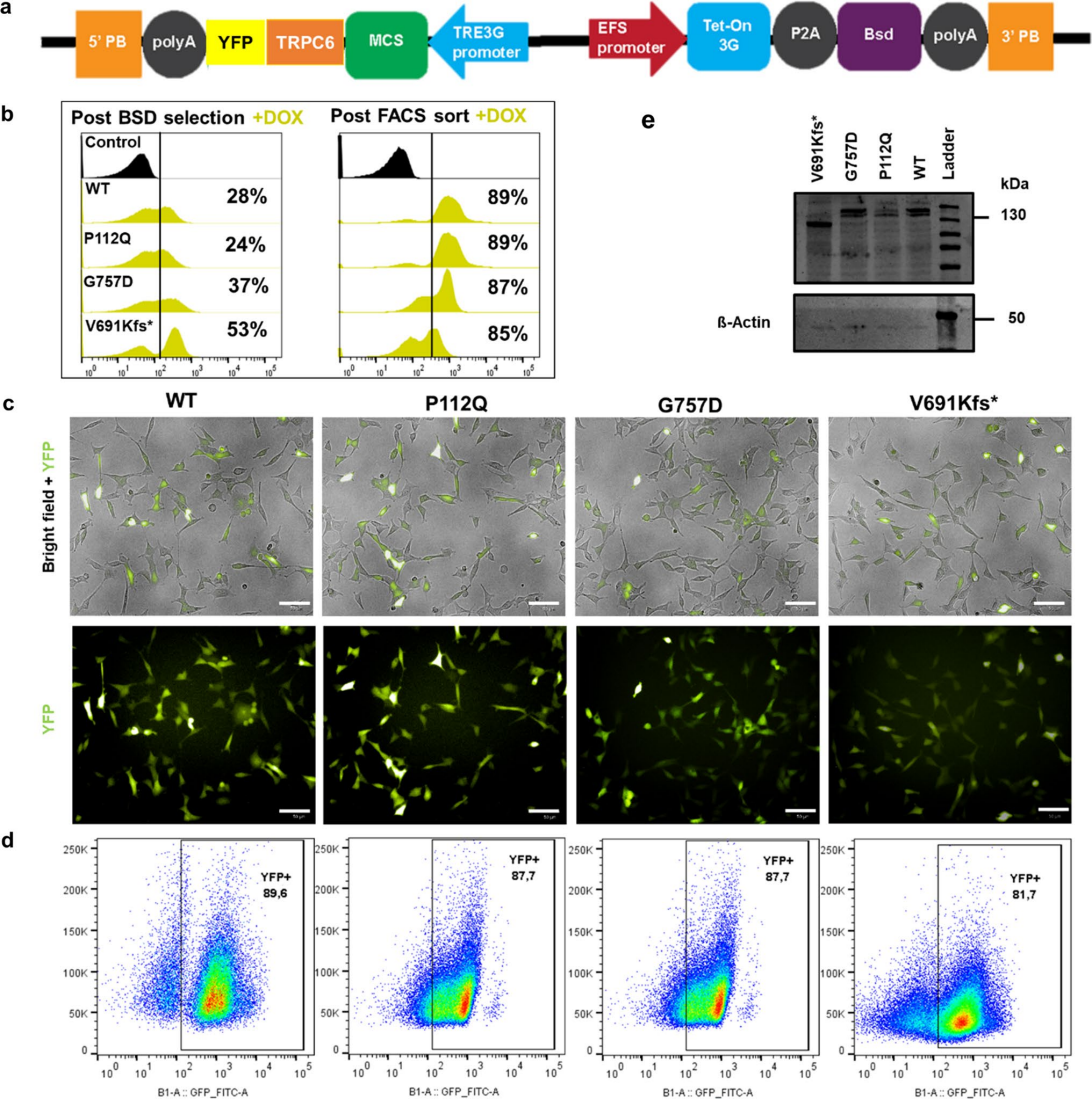
Design and generation of XLone transgenic HeLa cells. **a** Schematic design showing the transposable plasmid cassette (*5′ PB* 5′ PiggyBac Terminal Repeat; *3′ PB* 3′ PiggyBac Terminal Repeat; *MCS* multiple cloning sites; *Bsd* blasticidin resistance gene; *EFS* EF1 alpha promoter; *TRE3G* tetracycline responsive element 3G promoter). **b** Flow cytometry analysis of the cells after Bsd drug selection and FACS-live cell sorting of respective wild type, P112Q, G757D, and V691Kfs*. **c** Representative images illustrate the expression of YFP reporter (green) after transfection, Bsd selection, and FACs sorting, as shown by the merged image of the bright field and YFP signal for each of the variants studied. Scale bar 50 µm. **d** Representative FACS plots of transgenic HeLa cells demonstrate an enriched population up to a maximum of 89% YFP-positive cells. **e** Western blot for TRPC6 protein from wild-type, p112Q, G757D, and V691kfs* (~ 130 kDa) (B) β-actin (~ 45 kDa) in HeLa cells

After transfection and blasticidin (Bsd) selection in HeLa cells, we observed a heterogeneous population of cells expressing YFP signals reaching between 28% to 53% (Fig. 3b). However, FACS sorting after dox induction alone without Bsd selection yielded more TRPC6/YFP-positive cells (Fig. 3b–d). Therefore, we used FACS-live sorting for the purification of the cells responsible for dox-inducible TRCP6/YFP expression. The expression of TRPC6 protein in HeLa cells was detected by Western blotting, and the results confirmed that TRPC6 protein was expressed in those cells carrying TRPC6 variants. The data showed the fact that wild-type and mutant V691Kfs* differ sufficiently in molecular weight in order of their relative expression levels (Fig. 3e).

### Calcium influx studies indicate that the V691Kfs* is non-functional

**Fig. 4:**
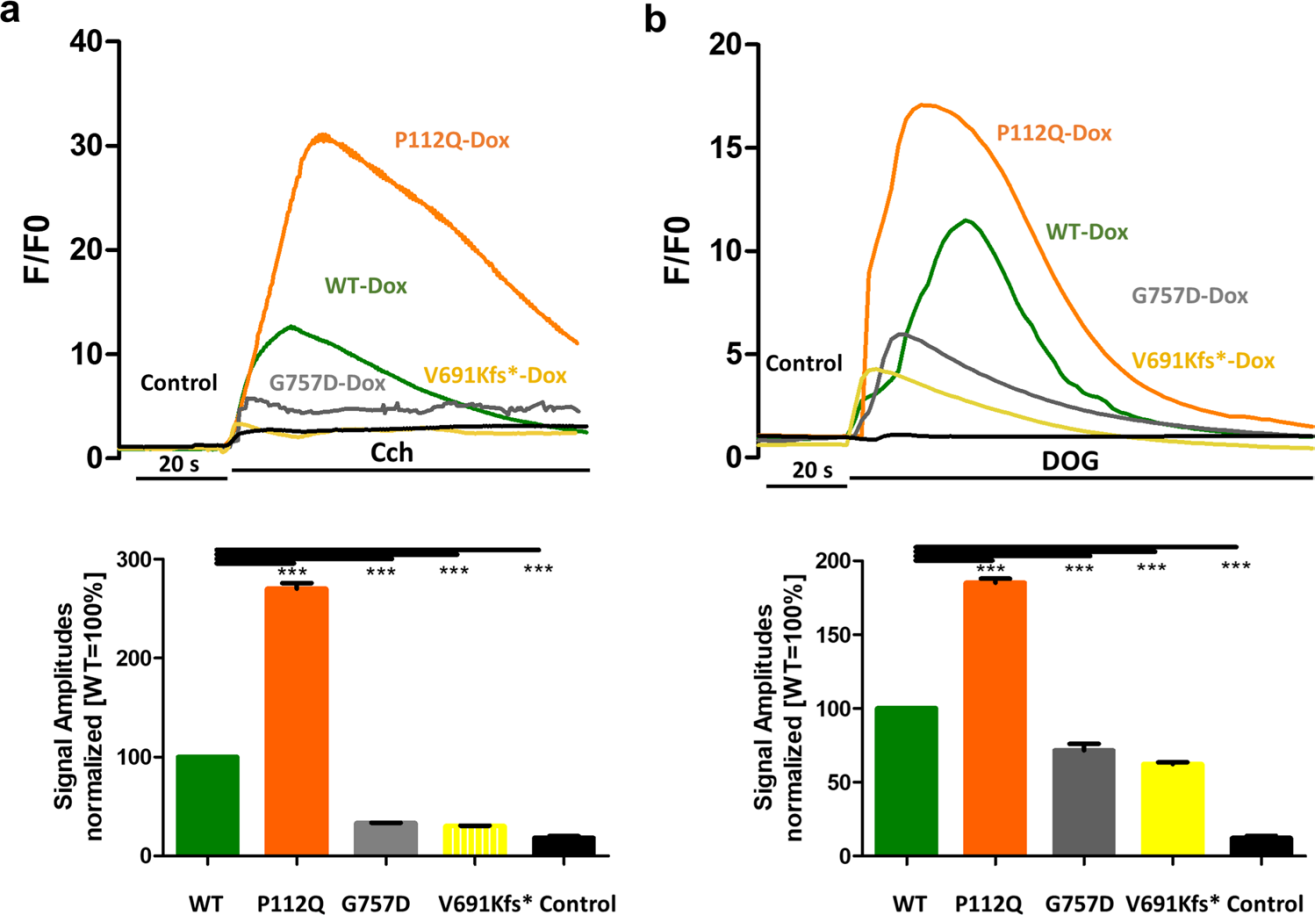
Functional characterization reveals GOF, LOF, and truncated phenotype in FSGS-related TRPC6 mutations. Changes in intracellular Ca^2+^ concentration (*F*/*F*_0_) were measured in Fluo-4 AM-loaded HeLa cells pretreated with doxycycline (Dox) (24 h prior) expressing either wild type (WT), P112Q, G757D, or V691Kfs*. Each condition was carried out in replicates, while un-transfected HeLa cells served as control. **a** Carbachol (Cch) (100 µM) was added 20 s after the start of the measurement. Representative measurement for all samples is shown in the upper panel, while a summary is presented as a bar graph (*n* = 3, mean ± SEM) in the lower panel. The graph shows the differences in amplitudes of the responses to Cch. **b** DOG (100 µM) was added 20 s after the start of the measurement. Representative measurement for all samples is shown in the upper panel, while a summary is presented as a bar graph (*n* = 3, mean ± SEM) in the lower panel. The graph shows the differences in amplitudes of the responses to DOG

**Fig. 5:**
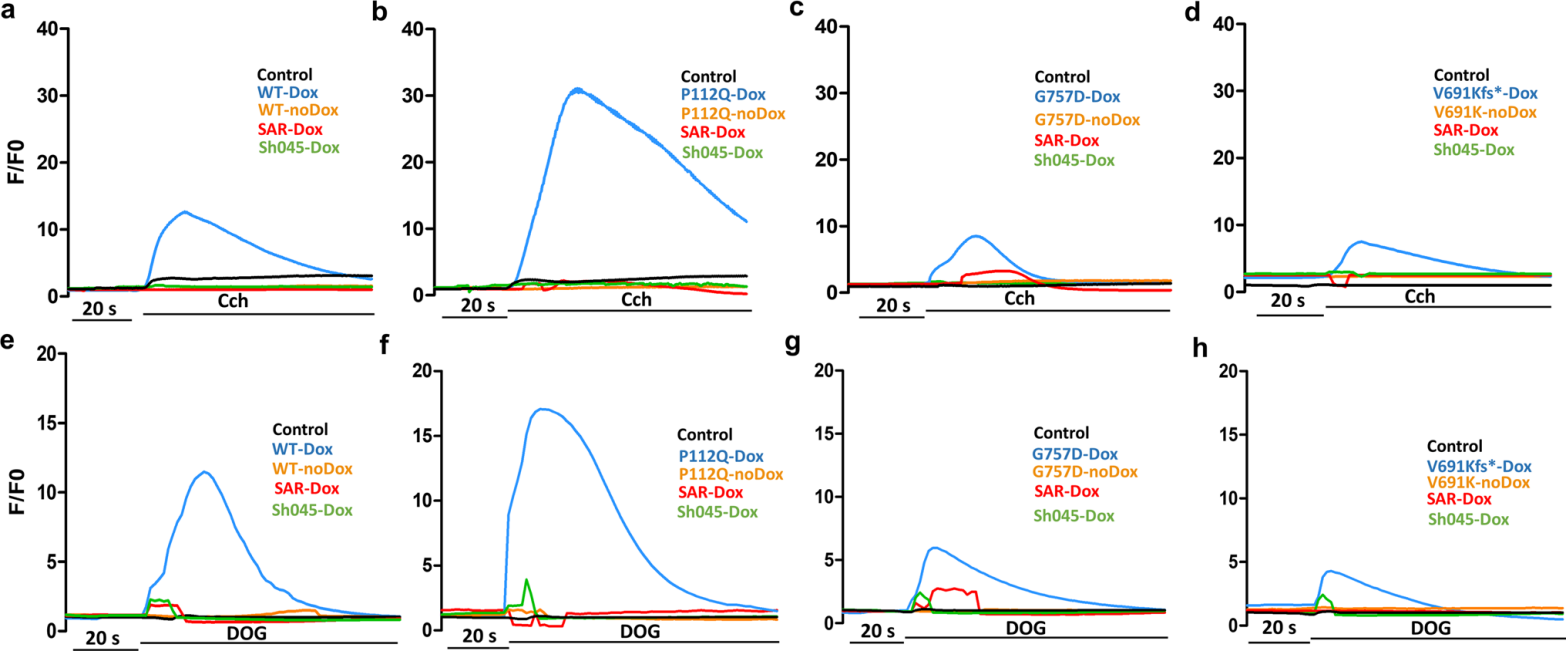
Functional characterization of disease-related TRPC6 mutants in calcium influx measurements. Changes in intracellular calcium concentration (*F*/*F*_0_) were measured in Fluo-4-AM-loaded HeLa cells expressing wild type (WT), P112Q, G757D, or V691Kfs* treated with doxycycline (Dox) (0.5 µg/mL) 24 h before measurement and no Dox treated cells. **a**–**d** Treatment with agonist carbachol (Cch: 100 µM): Cch was added about 20 s after the start of the measurement. Effects of TRPC6 blockers SAR7334 (SAR; 100 µM) and SH045 (100 µM) are observed in each TRPC6 mutant. **e**–**h** DOG (100 µM) was added about 20 s after the start of the measurement. Effects of SAR7334 (SAR; 100 nM) and SH045 (100 nM) are observed in each TRPC6 mutant

The discovery of the new truncated mutation and the availability of known disease-related TRPC6 mutations allowed us to comprehensively study their functional significance in a novel cellular system. In the first set of experiments, we measured TRPC6 activity in response to carbachol [8]. TRPC6 P112Q showed increased Ca^2+^ influx compared to TRPC6 wild type, whereas TRPC6 G757D showed decreased Ca^2+^ influx in comparison to TRPC6 wild type and P112Q (Fig. 4a), which would be consistent with GOF and LOF phenotypes, respectively. Interestingly, the new truncated mutant TRPC6 V691Kfs* showed a reduced Ca^2+^ influx in response to carbachol (100 µM) compared to TRPC6 wild-type, P112Q, and G757D mutants (Fig. 4a). Based on the measurements from basal Ca^2+^ concentration and carbachol-induced amplitudes, the phenotype of the mutants seems to be heterogeneous. To further investigate that matter, we measured TRPC6 activity in response to a direct TRPC6 channel activator, 1,2-dioctanoyl-sn-glycerol (DOG), to measure Ca^2+^ influx. TRPC6 P112Q showed increased Ca^2+^ signal amplitudes, consistent with the GOF phenotype (Fig. 4b) and TRPC6 G757D responded to DOG with a significantly reduced signal amplitude, consistent with the LOF phenotype (Fig. 4b). TRPC6 V691Kfs* showed the lowest response to DOG (Fig. 4b), which can be distinguished from the wild-type, P112Q, and G757D mutants. The limited response of V691Kfs* against carbachol and DOG supports the inactivity of the TRPC6 channel arguing for the closure of the ion transport pore. Part of the remaining Ca^2+^ influx in TRPC6 V691Kfs*-expressing cells may have been caused by the tetracycline-control gene regulatory system used for TRPC6 expression that might have resulted due to uncontrolled Tet promotor to express the transgene after dox induction [33]. Furthermore, we measured the response of wild-type, P112Q, G757D, and V691Kfs* mutants against TRPC6 channel inhibitors, SAR7334 (100 nM)[30] and SH045 (100 nM) [34], that have been reported previously to show high selectivity, particularly toward TRPC6 and also TRPC3 and TRPC7. The data show that 10-min pre-treatment of TRPC6 mutant-expressing cells with SAR7334 and SH045 individually inhibits TRPC6-mediated Ca^2+^ influx when stimulated with carbachol and DOG (Fig. 5a–h), which indicates that both the antagonists are specific for the TRPC6 channel.

**Fig. 6:**
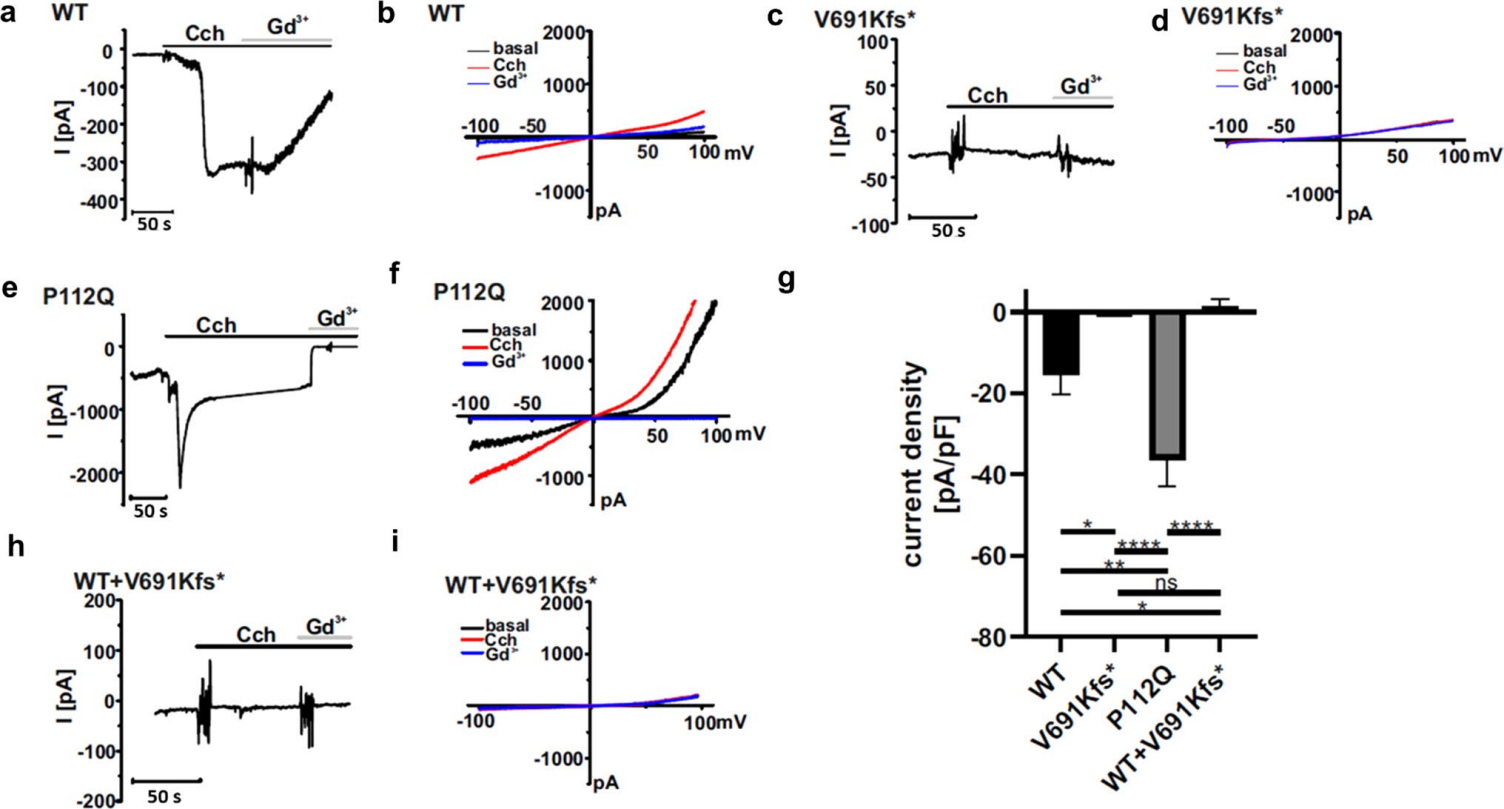
Electrophysiologic characterization revealed currents mediated by WT TRPC6, TRPC6 V691Kfs*, TRPC6 P112Q, and TRPC6 WT + V691Kfs*. Whole-cell currents were recorded in transfected HEK293 cells using the protocol described in the “Materials and methods” section. After obtaining the whole-cell configuration, currents from −100 to + 100 mV were recorded during voltage ramps. **a**, **c**, **e**, **h** Inward currents at −60 mV were plotted over time. **b**, **d**, **f**, **i** Current–voltage relationships obtained during voltage ramps from −100 to + 100 mV: 1, basal currents (black); 2, 100 µM carbachol (Cch)-induced currents (red); and 3, 100 µM Cch-induced currents in the presence of 100 µM gadolinium chloride (Gd^3+^, blue). **a**, **b** Currents were measured in WT TRPC6 cells. **c**, **d** Currents were recorded in TRPC6 V691Kfs*-transfected cells. **e**, **f** Currents were recorded in TRPC6 P112Q-expressing cells. **h**, **i** Currents were recorded in TRPC6 WT + V691Kfs*-transfected cells. **g** Statistical analysis of the Cch-activated currents (plotted as the current density at −60 mV). Data are means ± SEMs in each group: six cells (WT TRPC6, black), seven cells (truncated TRPC6), eight cells (TRPC6 WT + V691Kfs*), and five cells (TRPC6 P112Q, gray). *P ≤ 0.05; **P ≤ 0.01; ****P ≤ 0.0001

To more directly explore the difference between TRPC6 wild-type, V691Kfs*, and P112Q mutants, we recorded whole-cell currents in transfected HEK293 cells [19]. Whole-cell membrane currents with distinctive inward and outward rectification were activated by carbachol (100 µM) in wild-type TRPC6-expressing cells (Fig. 6a, b). The carbachol-induced current was inhibited by gadolinium chloride (Gd^3+^, 100 µM) (Fig. 6a, b). In contrast, carbachol was unable to induce Gd^3+^-sensitive TRPC6 currents in HEK293 cells transfected with TRPC6 V691Kfs* (Fig. 6c, d). We validated our findings by recording whole-cell currents in cells transfected with P112Q, which has been previously described as GOF [19]. Consistent with our previous findings [19], whole-cell currents in response to carbachol were largely increased in P112Q-expressing cells (Fig. 6e, f). However, there were almost no carbachol current amplitudes observed in V691Kfs*-expressing cells as compared to wild-type and P112Q current amplitudes, confirming the non-functionality of the TRPC6 channel (Fig. 6g). Statistical analysis shows that the WT/V691Kfs* and WT/P112Q differences are significant. Carbachol-induced whole-cell currents obtained in co-transfected wild-type/V691fs*-expressing cells were negligibly small (Fig. 6h, i) and could be clearly distinguished from whole-cell currents obtained in wild-type and P112Q-expressing cells (Fig. 6b, f), which indicates non-functionality of the TRPC6 channel. The data indicate a dominant negative effect when co-expressed with WT TRPC6. Statistical analysis is shown in Fig. 6g.

### Co-expression shows a dominant negative effect

**Fig. 7:**
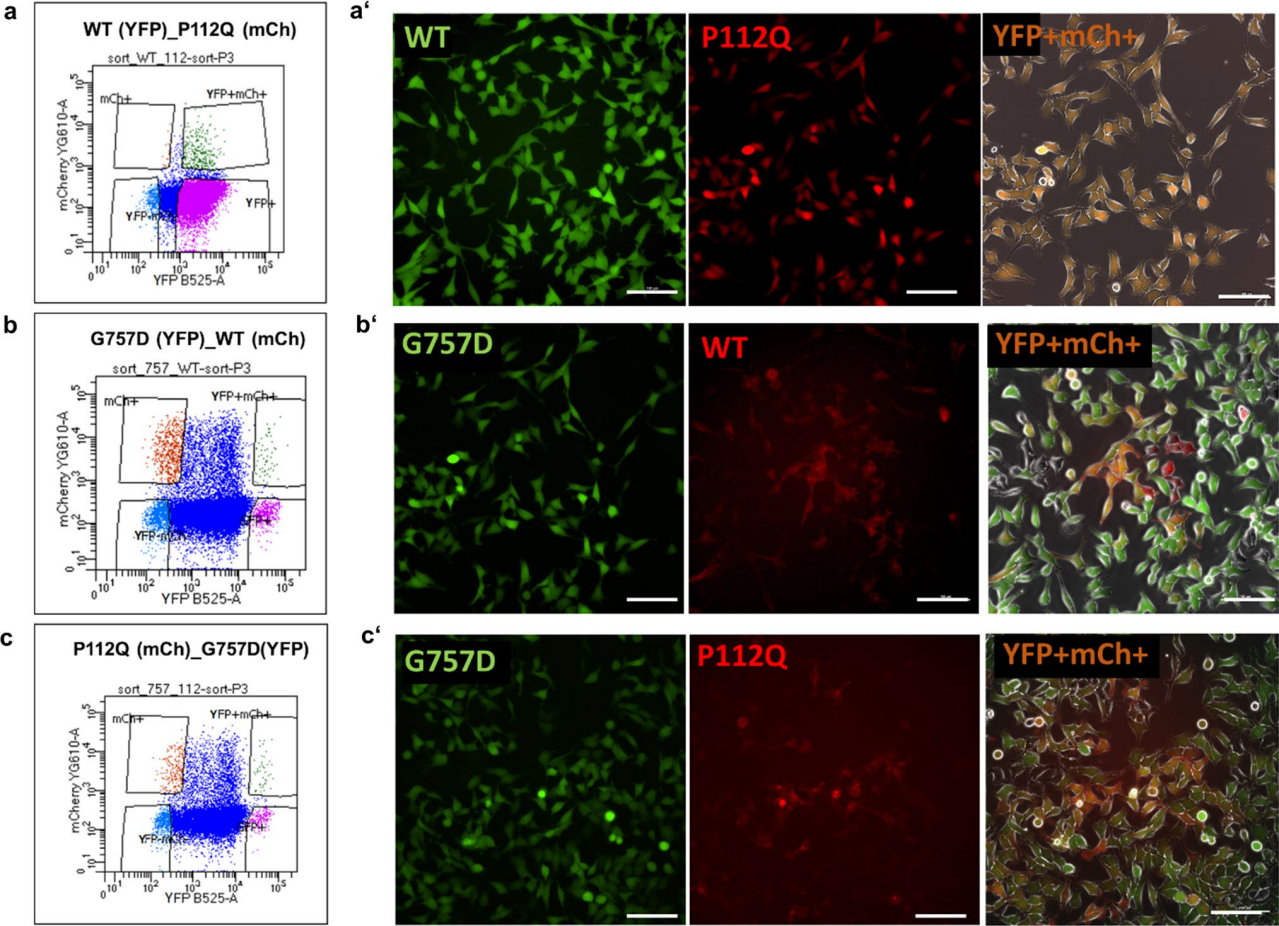
Co-transfection strategy to characterize the dominant phenotype of TRPC6 mutants. Cells were transfected with mixtures of TRPC6 Wild Type (WT) C-terminally fused to YFP and mCherry, G757D C-terminally fused to YFP and P112Q fused to mCherry (mCh +). **a**–**c** Representative flow cytometry plots show the gating strategy to sort double-positive cells expressing both YFP and mCherry (YFP + mCh + , top right) for each co-transfected cells and the remaining 3 gates to sort cells expressing only mChr + (Top left), YFP (bottom right), and non-transfected cells (bottom left). Microscopic images of (**a**′) sorted cells expressing both WT-YFP/P112Q-mCh represented in combined image as YFP + mCh + , (**b**′) sorted cells expressing both G757D-YFP/WT-mCh represented in combined image as YFP + mCh + , (**c**′) sorted cells expressing both G757D-YFP/P112Q-mCh represented in combined image as YFP + mCh + . Scale bar 100 µm

**Fig. 8:**
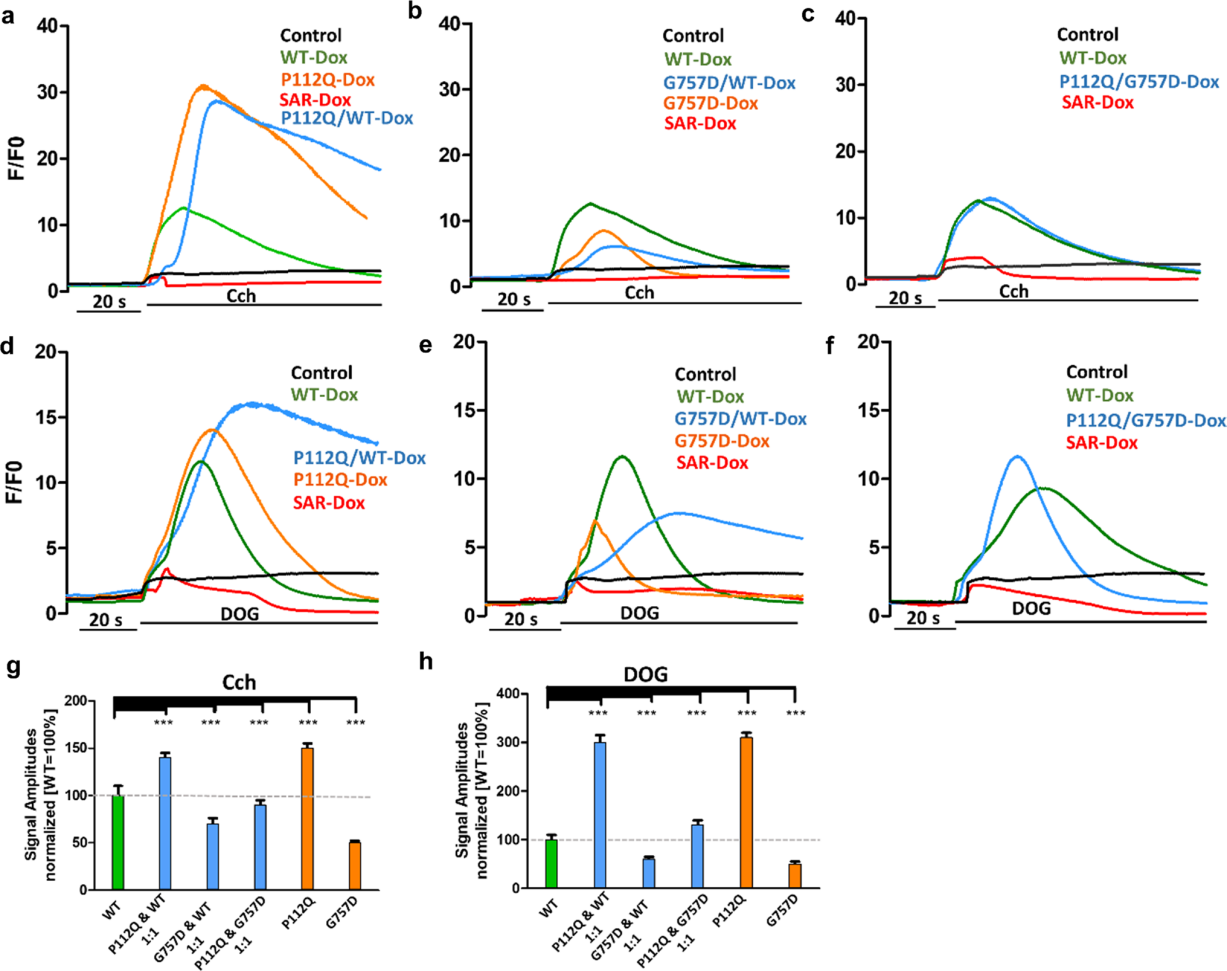
Co-transfection strategy to characterize the dominant phenotype of TRPC6 mutants. Changes in intracellular calcium concentration (*F*/*F*_0_) were measured in Fluo-4-AM-loaded HeLa cells expressing wild-type (WT), P112Q/WT, G757D/WT, or P112Q/ G757D TRPC6 C-terminally fused to YFP and mCherry treated with doxycycline (Dox) (0.5 µg/mL) 24 h before measurement. **a**–**c** Carbachol (Cch: 100 µM) was added about 20 s after the start of the measurement. Effects of SAR7334 (SAR; 100 µM) and SH045 (100 µM) are observed in each TRPC6 mutant. **d**–**f** DOG (100 µM) was added about 20 s after the start of the measurement. Effects of SAR7334 (SAR; 100 µM) and SH045 (100 µM) are observed in each TRPC6 mutant. **g** Statistical analysis of data obtained from at least three independent experiments is presented as bar graphs. The graphs show the differences in amplitudes of the responses on Cch application **h** Statistical analysis of data obtained from at least three independent experiments is presented as bar graphs. The graphs show the differences in amplitudes of the responses to the DOG application (*n* = 3, mean ± SEM). ***P ≤ 0.0001

To determine the dominant phenotypic characteristics of TRPC6 mutants that are consistent with the heterozygosity of TRPC6 in vivo, we performed in vitro experiments to categorize the effect of P112Q and G757D individually in a channel complex composed of wild-type and TRPC6 mutants. For this purpose, we studied carbachol and DOG-induced Ca^2+^ response in HeLa cells expressing wild-type and TRPC6 mutants in a 1:1 ratio (Fig. 7). To monitor equimolar expression, we used mCherry and YFP fusion constructs (Fig. 7). Cells transfected with P112Q/WT DNA mixture, pretreated with doxycycline, responded to carbachol with increased channel activity (Fig. 8a). Quantitative analysis revealed that cells transfected with the P112Q/wild-type mixture responded with an approximately two-fold increase of the response to the wild-type TRPC6, which is almost similar to the effect of P112Q alone (Fig. 8g). The same mixture of cells responded to DOG with a 50% increase in calcium response than the wild-type channel as seen for the effect of GOF alone (Fig. 8h). The 1:1 ratio of TRPC6 wild type and P112Q corroborates that in each tetrameric complex, at least one P112Q protein is integrated. Considering that the mutant affects only the function of one channel protein within the tetrameric complex, the mixture would result in an increased amplitude of effect on the overall channel activity, making a dominant GOF effect. Statistical analysis shows that DOG calcium amplitudes are smaller than carbachol-induced amplitudes; however, the wild-type/mutant differences are statistically significant.

In line with our previous findings [19], cells transfected with TRPC6 G757D/wild-type mixture showed reduced responses to the carbachol (Fig. 8b) and DOG (Fig. 8e), while their results are comparable with G757D alone. Statistical analysis revealed that the G757D/wild-type mixture responded to DOG and carbachol with about 40% of the response of the wild-type TRPC6, which is almost similar to G757D alone (Fig. 8g, h), thereby suggesting that the LOF response is lower than the TRPC6 wild-type response; however, such a low response is not sufficient to make an impact on the functionality of the channel in comparison to GOF phenotype showing a higher response. Likewise, cells transfected with P112Q/G757D together after doxycycline treatment showed a similar response as the wild type when treated with carbachol (Fig. 8c, g) and DOG (Fig. 8f, h). Hence, we assume that the mixture of both the GOF/LOF TRPC6 mutants restores the wild-type activity and the existence of a negative-dominance phenomenon with genetic variants of the renal channel TRPC6.

## Discussion

We describe and characterize a novel TRCP6 variant V691Kfs* in a large kindred exhibiting no signs of FSGS-related pathology. The rapid onset and progression of renal disease in the index patient is not typical of TRPC6-FSGS renal pathology, nor are the histological findings on renal biopsy. In some cases, the finding of MCD is due to unsampled FSGS, and in other instances, it may represent an earlier clinical stage of FSGS, which is unlikely in the present case. Also, although the pedigree is relatively large, only two family members (one with an unknown genotype) had renal disease, which is atypical for an autosomal dominant inheritance (even with less than complete penetrance). Finally, the pattern of the TRPC6 variant and inheritance does not follow a pattern of cosegregation (even when taking into account the age differences of the mutation carriers). The heterozygous TRPC6 frameshift mutation encodes a truncated TRPC6 protein lacking the intracellular cytosolic C-terminal tail, a highly conserved domain that plays an essential role in the TRPC6 channel activity, together with an exchange of valine to lysine at amino acid position p.691. The three-dimensional TRPC6 structure shows that V691Kfs* is located at the pore opening region of the tetrameric channel complex. The truncation introduced by V691Kfs* is predicted to cause a complete closure of the ion-conducting pore resulting in a dominant downstream LOF effect. To substantiate this hypothesis, the expressed mutant showed channel activity below the activation level of wild-type TRPC6, even lower than the previously described G757D LOF mutation, arguing for an inactive TRPC6 channel. In addition, the V691Kfs* truncation exerted a dominant negative effect on the full-length TRPC6 proteins. The pedigree analysis of kidney disease in the index family showed that albeit the presence of the truncated FSGS—mutant across generations, kidney function is normal. This finding together with our in vitro cell data thus argues against the loss of TRPC6 function as a generalized concept of hereditary FSGS in humans [19].

In the kidney, TRPC6 is expressed in renal tubules and predominantly in podocytes of the glomeruli. It consists of four ankyrin repeats at the amino (N)-terminal, six transmembranes (TM) domains with a pore formation at TM 5 and 6 for ion transport, and a carboxyl (C)-terminal [35]. Multisequence alignment studies revealed that all the detected TRPC6-related mutations reside in these highly conserved sites and the in silico approach depicts non-tolerated changes that affect protein function. One mutation (P112Q) described here is located in the ankyrin repeats at N-terminal and one mutation (G757D) is in the C-terminus. There are 10 different mutations [14–16, 22] reported so far that have affected the same domains, thereby highlighting the essential roles of these domains for protein function. The novel p.V691Kfs* TRPC6 mutation identified in this study is the first mutation located in the extracellular or pore-forming transmembrane (TM) segment 6, region of the channel. Although a majority of the mutations in the N/C-terminal tails are more susceptible to acquiring the disease phenotype, the depicted mutation in the TM-6 does not lead to any clinical (renal) phenotype. We conclude that the LOF TRPC6 character is not a direct cause of FSGS in humans. Our co-expression experiments of different disease-causing mutations revealed the existence of a negative-dominance phenomenon with genetic variants of the renal channel TRPC6. This phenomenon is known for many genetic variants of various multimeric cardiac K^+^, Ca^2+^, Cl^−^, and Na^+^ channels, and in particular, Brugada syndrome variants of Na_v_1.5 [36]. For example, epidemiological studies have demonstrated that patients that carry KCNQ1 missense mutations with a dominant negative effect have a poorer outcome than individuals with nonsense mutations that lead to haploinsufficiency [37]. Furthermore, if such distinct heterogeneous ion channel complexes exist in renal TRPC channels, it would be interesting to determine whether a dominant negative mutant of one type of channel can affect the activity of another type of channel that affects the renal outcome. In this regard, our data support the novel concept that inhibition of TRPC6 channels, possibly cross-talking with TRPC3 [38], represents a promising new therapeutic approach to ameliorate renal stress-induced disease with fibrosis [31, 39–43].

To date, two C-terminal truncated TRPC6 mutations (K874X and D873fsX878) have been identified in FSGS patients [9, 22, 44, 45]. The frameshift TRPC6 mutation (D873fsX878) is predicted to result in a truncated TRPC6 protein lacking the C-terminal coiled-coil domain. This mutation is likely to have a dominant GOF effect similar to other known pathogenic TRPC6 mutations mapped to the coiled-coil domain at the C-terminus, for example, R895C and E897K [22]. The other truncated TRPC6 mutation (K874X) encodes a premature stop codon near the C-terminus [9]. This mutation does not produce changes in the TRPC6 peak current amplitudes but reduces calcium channel inactivation, consistent with a GOF phenotype [20, 46]. In our study, the V691Kfs* mutant is mapped at the transmembrane domain S6, lining the pore lumen of the TRPC6 channel. The truncated mutant results in the removal of the S6 domain near the C-terminus disrupting the pore helix and pore loop interactions with other linker domains. The Ca^2+^ imaging experiments and whole-cell electrophysiological recordings on the V691Kfs* mutant demonstrated a non-functional, fully inactivated TRCP6 channel, which is consistent with a complete LOF phenotype. In contrast, the functional characterization of TRPC6 P112Q in recombinant expression systems depicted that replacement of these amino acids causes increased TRPC6 activity in our expression system by showing a two-fold increase in calcium influx in comparison to other TRPC6 variants [9, 14].

To date, animal models recapitulating autosomal dominant forms of FSGS have not been reported because they failed to produce impressive glomerular pathology. Studies in multiple strains of mice and rats with global knockouts or complete loss of TRPC6 function also exhibit no signs of nephrosis, even in old age [41]. Kim and Dryer observed that the loss of functional TRPC6 channels in rats neither enhanced nor deteriorated the overall renal function that usually occurs with age and did not consistently reduce any inflammation or tubulointerstitial fibrosis [41]. Indeed, as noted, several studies indicate that complete loss of TRPC6 can be protective in a variety of kidney disease models. TRPC6 knockout in the chronic PAN model in rats substantially reduced the severity of tubulointerstitial disease based on histological analysis and measurements of biochemical markers of inflammation and fibrosis as compared to rats with wild-type TRPC6 [42]. A significant protective effect of TRPC6 inactivation on the severity of glomerular damage (glomerulosclerosis) was ascertained by semi-quantitative analysis of light microscopic images [47]. Likewise, TRPC6 knockout has been shown to have protective effects in mouse models in which tubulointerstitial disease is followed from an initial insult to tubules as a result of unilateral ureteral obstruction (UUO) [40]. On the other hand, our in vitro data and the index patient who showed partial remission of renal disease (MCD) on cyclophosphamide and had 22 pedigree members without the renal disease (which is not typical for a monogenic autosomal dominant kidney disease) support the notion that loss of function does not inevitably lead to renal disease. Furthermore, transgenic overexpression of either wild-type or mutant *TRPC6* (P111Q and E896K GOF) in podocytes causes only modest albuminuria and mild histological changes with incomplete penetrance in mice [48]. Human autosomal dominant FSGS-causing mutations in *TRPC6* [49], *ACTN4* [50], and *INF2* [51] are unable to induce a renal phenotype when present in heterozygous mice. Although homozygous *Actn4* or *Inf*2 mutant mice show enhanced sensitivity to injury [50, 52], this is not observed in homozygous mutant TRPC6 (E896K GOF) mice exhibiting no susceptibility to PAN nephrosis [49]. The reason for the relative lack of a renal phenotype in the TRPC6 knock-in/-out animals remains unclear. Reports of differences in TRPC6 mRNA expression between mouse strains [53] and strain-specific susceptibility to kidney and glomerular injury are well known. Uncertainty exists on whether TRPC6 mutations need a genetic or environmental trigger to induce glomerular disease in mice, or if mice are intrinsically not suitable to model TRPC6-mediated human FSGS.

TRPC6 has been shown to interact with several proteins in the podocyte, including podocin and nephrin [9]. This led us to wonder whether there are other, apart from calcium conductance, TRPC6 protein interactions that are important in podocyte function. The study from Farmer et al. clearly showed that proteolytic turnover of focal adhesion proteins involving calpains is an important driver in the pathogenesis of FSGS [45]. Calpain activity was significantly downregulated in TRPC6 knockout podocytes in a similar fashion as in the calpain inhibitor calpeptin-treated control and TRPC6 wild-type cells. Importantly, the regulation of calpain activity by TRPC6 was shown to be independent of alterations in its calcium conductance [45]. Interestingly, more recent reports show that TRPC6 activity is linked to increased calpain 1 and calcineurin activity contributing to PAN-induced podocyte injury [54] as well as promoting the pathogenesis of nephrotic syndrome and podocyte injury [55, 56]. These studies present a puzzling anomaly regarding the studies from Riehle et al. [19] showing FSGS-related disease mutant G757D-mediated changes of TRPC6 activity.

*TRPC6* mutations, although more rare with regard to the number of affected families, anticipate crucial novel clues toward the understanding of hereditary forms of proteinuric glomerular diseases. Several studies showed that TRPC6 expression is upregulated in various human proteinuric kidney diseases, such as minimal change disease (MCD), membranous glomerulonephritis (MGN), autoimmune glomerulonephritis, diabetic kidney disease (DKD), and FSGS [42, 57–59]. Meanwhile, in vitro and in vivo models of glomerular diseases have also shown induced levels of TRPC6 protein in podocytes. All these findings are in line with increased TRPC6 channel activity in various proteinuric kidney diseases, indicating detrimental effects of hyperactive TRPC6 activity, whether it stems from mutations, environmental factors, risk factors, or drugs. Our data support the idea that inhibition of TRPC6 channels may constitute a therapeutic strategy for improving renal outcomes [31, 39–43].

In conclusion, we identified a new truncated TRPC6 p.V691fs* mutant mediating TRPC6 channel inactivation in a large pedigree exhibiting no signs of FSGS. Our results show that loss of TRPC6 channel function is unlikely to be solely responsible for early or late onset FSGS. The data emphasize the need to further investigate TRPC6 channel mechanisms and interactions with other podocyte proteins in the pathogenesis of human FSGS.

### Supplementary Information

Below is the link to the electronic supplementary material.

Supplementary file1 (DOCX 596 KB)

## Data Availability

All data generated or analyzed during the present study are included in the present article.
