# Supplementary material for: An inactivating human TRPC6 channel mutation without focal segmental glomerulosclerosis
Source: Cell Mol Life Sci. 2023 Aug 24;80(9):265. doi: 10.1007/s00018-023-04901-w (PMC10449997; doi:10.1007/s00018-023-04901-w)
Supplement: Supplementary file 1 — Supplementary file1 (DOCX 596 KB) [file 18_2023_4901_MOESM1_ESM.docx]

**Supplementary Table S1: List of sequence variants for each TRPC6 mutant and primer sequence**

| **Plasmid Sequence variant 5’ → 3’** | |
| --- | --- |
| TRPC6-WT-YFP | Sequence from (Addgene#21084) |
| TRPC6-P112Q-YFP | ctttttggatgcagctgaatatggtaaTatTcAagtggtgcggaagatgttagaaga |
| TRPC6-G757D-YFP | tctggttttcctactttgaggaggAcagaacacttcctgtacccttcaatct |
| TRPC6-V691Kfs* | ttctgggctatatttggactttctgaagtgaaatcaAAGgtga |
| **Sequencing** | |
| **Primer name Sequence 5’ → 3’** | |
| TRPC6_P112Q_seq_fwd | TGATCGCTCCACAAGCCTATC |
| TRPC6_G757D_seq_fwd | CTACTCCTACTACATTGGTGC |
| M13-rev | CCCAGTCACGACGTTGTAAAACG |
| TRPC6_seq_rv | CTGCCAACTGTAGGGCATTCT |


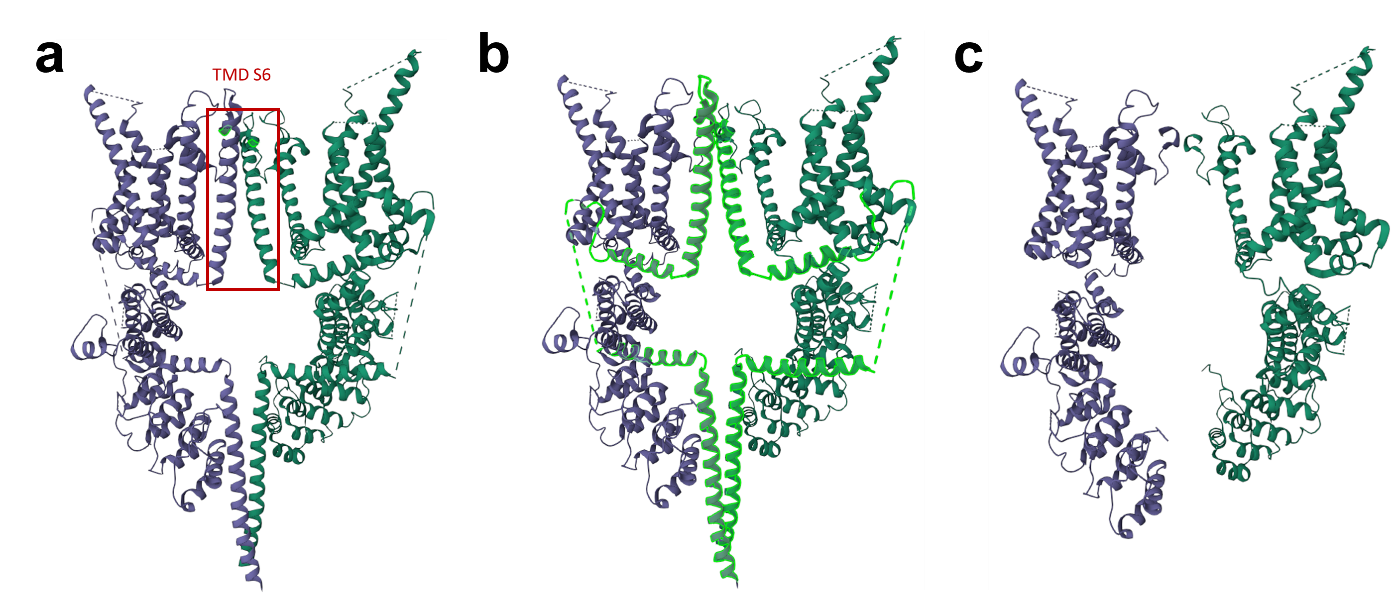


**Supplementary Figure 1:** Tridimensional Cryo-EM structure of tetrameric TRPC6 (PDB: 6UZ8). **a)** Two subunits of wild-type TRPC6 indicating the transmembrane domain (TMD) S6 with highlighted position of V691Kfs* in bright green. **b)** The truncation results in the removal of the highlighted helices. **c)** Truncated TRPC6 channel with loss of TMD S6.
